# Supplementary material for: The rise in stunting in relation to avian influenza and food consumption patterns in Lower Egypt in comparison to Upper Egypt: results from 2005 and 2008 Demographic and Health Surveys
Source: BMC Public Health. 2015 Mar 25;15:285. doi: 10.1186/s12889-015-1627-3 (PMC4405853; doi:10.1186/s12889-015-1627-3)
Supplement: Additional file 1: — The association between child stunting and water and sanitation variables in bivariate and multivariable analyses, Lower and Upper Egypt, 2005 and 2008 EDHS. Bivariate and multivariable analyses of child stunting and water and sanitation variables using 2005 and 2008 EDHS data. Regression models presented in main manuscript, are presented in this file, including water and sanitation variables. [file 12889_2015_1627_MOESM1_ESM.pdf]

Additional file: The association between child stunting and water and sanitation variables, in bivariate and multivariable analyses, Lower and Upper Egypt, 2005 and 2008 EDHS

Table 1. Proportion of stunting, 6-59 months, Lower Egypt 2005 (N= 2,292) and 2008 (N = 2,293)

Factor	2005			2008		
	Total N [%]	Stunting, 6-59 months n [%]	p-value	Total N [%]	Stunting, 6-59 months n [%]	p-value
**Drinking Water Source (Type)**						
Piped Water	2176 [94.94]	367 [16.87]	0.176	2143 [93.46]	671 [31.31]	0.493
**Toilet Facility**						
Modern Flush Toilet	846 [36.91]	134 [15.84]	0.441	1020 [44.48]	341 [33.43]	0.073

Table 2. Proportion of stunting, 6-59 months, Upper Egypt 2005 (N= 3,893) and 2008 (N = 2,708)

Factor	2005			2008		
	Total N [%]	Stunting, 6-59 months n [%]	p-value	Total N [%]	Stunting, 6-59 months n [%]	p-value
**Drinking Water Source (Type)**						
Piped Water	3577 [91.88]	1021 [28.54]	0.294	2527 [93.32]	548 [21.69]	0.633
**Toilet Facility**						
Modern Flush Toilet	564 [14.49]	107 [18.97]	**<0.001**	530 [19.57]	15.85 [84]	**<0.001**

Table 3: Determinants of stunting, children 6-59 months, Lower Egypt 2005 (N= 2,292) and 2008 (N= 2,293)

**Factor**	**Adjusted OR 2005**	**P-value**	**95% CI**	**Adjusted OR 2008**	**P-value**	**95% CI**
**Residence**						
Urban (ref)	-	-	-	-	-	-
Rural	0.962	0.839	0.659, 1.403	0.990	0.958	0.678, 1.446
**Age of child**						
6-11 months (ref)	-	-	-	-	-	-
12-23 months	0.838	0.451	0.528, 1.333	2.267	**<0.001**	1.519, 3.383
24-35 months	1.001	0.974	0.609, 1.670	2.122	**0.001**	1.390, 3.238
36-47 months	0.458	**0.006**	0.264, 0.794	2.339	**<0.001**	1.502, 3.643
48-59 months	0.377	**0.003**	0.196, 0.734	1.493	0.096	0.931, 2.393
**Sex of child**						
Females (ref)	-	-	-	-	-	-
Males	1.029	0.836	0.781, 1.357	1.289	0.023	1.036, 1.602
**Wealth Quintile**						

Poorest (ref)	-	-	-	-	-	-
Poorer	0.856	0.570	0.501, 1.464	1.097	0.653	0.733, 1.642
Middle	0.815	0.458	0.474, 1.401	1.002	0.993	0.667, 1.505
Richer	0.672	0.256	0.338, 1.336	0.901	0.969	0.532, 1.524
Richest	1.328	0.471	0.612, 2.883	1.050	0.878	0.564, 1.955
**Perceived size of child at birth**						
Very small or Small	1.204	0.400	0.781, 1.856	1.201	0.343	0.823, 1.753
Average or Larger (ref)	-	-	-	-	-	-
**Birth Intervals**						
<24 months	1.146	0.546	0.736, 1.784	1.384	0.063	0.982, 1.950
24-35 months	1.142	0.534	0.750, 1.740	0.833	0.270	0.602, 1.153
36-47 months (ref)	-	-	-	-	-	-
48+ months	0.711	0.102	0.472, 1.070	1.009	0.950	0.760, 1.339
**Avian influenza**						
**Household ownership of poultry/birds**	1.046	0.801	0.736, 1.488	0.582	**0.001**	0.418, 0.812
**Water and Sanitation**						
Piped water	1.461	0.319	0.692, 3.083	1.007	0.973	0.660, 1.536
Modern Flush Toilet	0.208	0.199	0.444, 1.185	1.105	0.616	0.747, 1.635

Table 4: Determinants of stunting, children 6-59 months, Upper Egypt 2005 (N= 3,893) and 2008 (N= 2,708)

**Factor**	**Adjusted OR 2005**	**P-value**	**95% CI**	**Adjusted OR 2008**	**P-value**	**95% CI**
**Residence**						
Urban (ref)	-	-	-	-	-	-
Rural	1.119	0.540	0.780, 1.605	0.926	0.640	0.669, 1.281
**Age of child**						
6-11 months (ref)	-	-	-	-	-	-
12-23 months	1.449	**0.013**	1.082, 1.940	2.618	**<0.001**	1.728, 3.969
24-35 months	1.026	0.877	0.745, 1.412	1.651	**0.022**	1.075, 2.535
36-47 months	0.946	0.742	0.677, 1.320	1.571	**0.043**	1.015, 2.433
48-59 months	1.023	0.906	0.697, 1.502	1.841	**0.018**	1.112, 3.046
**Sex of child**						
Females (ref)	-	-	-	-	-	-
Males	1.226	**0.042**	1.007, 1.493	1.075	0.548	0.849, 1.361
**Wealth Quintile**						
Poorest (ref)	-	-	-	-	-	-
Poorer	0.587	**<.001**	0.452, 0.762	0.880	0.338	0.677, 1.143
Middle	0.594	**0.001**	0.437, 0.807	0.670	**0.023**	0.475, 0.945
Richer	0.697	0.174	0.414, 1.173	0.768	0.343	0.445, 1.326

Richest	1.044	0.922	0.440, 2.478	0.601	0.164	0.919, 1.513
**Perceived size of child at birth**						
Very small or Small	1.444	**0.007**	1.106, 1.884	1.468	**0.008**	1.108, 1.945
Average or Larger (ref)	-	-	-	-	-	-
**Birth Intervals**						
<24 months	0.967	0.812	0.733, 1.276	0.803	0.207	0.570, 1.130
24-35 months	1.043	0.767	0.790, 1.378	0.775	0.112	0.566, 1.062
36-47 months (ref)	-	-	-	-	-	-
48+ months	0.807	0.174	0.592, 1.100	0.668	**0.008**	0.496, 0.900
**Avian influenza**						
**Household ownership of poultry/birds**	0.933	0.507	0.761, 1.144	1.179	0.194	0.919, 1.513
**Water and Sanitation**						
Piped water	1.090	0.624	0.772, 1.540	0.997	0.987	0.661, 1.503
Modern Flush Toilet	0.395	**0.011**	0.193, 0.805	0.853	0.568	0.494, 1.474
